# Extremely Heavy Lifting Associated With Spontaneous Coronary Artery Dissection

**DOI:** 10.7759/cureus.19451

**Published:** 2021-11-10

**Authors:** Ellery Altshuler, Eldon Matthia, Dhaval Naik, Ellen C Keeley

**Affiliations:** 1 Internal Medicine, University of Florida College of Medicine, Gainesville, USA; 2 Cardiology, University of Florida College of Medicine, Gainesville, USA; 3 Department of Medicine, University of Florida College of Medicine, Gainesville, USA

**Keywords:** work-related injury, cardiac chest pain, weight lifting, scad management, atypical spontaneous coronary artery dissection

## Abstract

Spontaneous coronary artery dissection (SCAD) is a separation of the intimal wall by intramural hemorrhage and has been classically associated with young women. We report a case of a healthy 58-year-old man who presented with chest pain that arose shortly after lifting machinery that was known to weigh 200-250 pounds. He was admitted with a non-ST elevation myocardial infarction and was later found to have non-atherosclerotic SCAD. No underlying cause was identified, and the patient was managed medically. This case illustrates that physicians should maintain an index of suspicion for SCAD as a cause of acute coronary syndrome even in male patients without diseases associated with the condition, especially when heavy lifting is reported.

## Introduction

Non-atherosclerotic spontaneous coronary artery dissection (SCAD) is a separation of the coronary artery intimal wall by intramural hemorrhage [1]. Pressure-driven expansion of the hematoma within the lumen can restrict the passage of blood in the true lumen and thus cause cardiac ischemia [1]. Accordingly, clinical presentations of patients with SCAD are difficult to distinguish from other causes of cardiac ischemia. Although SCAD is commonly associated with young peripartum women with certain underlying conditions such as fibromuscular dysplasia, SCAD can occur in any population [1]. Most people with SCAD present with classic signs and symptoms of acute myocardial infarction; chest pain is present in 96% of cases [2]. The left anterior descending (LAD) is involved 60% of the time [3]. A review of 13 million patients with acute coronary syndrome (ACS) from 2005 to 2015 showed that SCAD was present in 0.49% of cases [4].

We report a case of a 58-year-old male without any underlying disease who presented with chest pain after repeatedly lifting machinery that ranged between 200 and 250 pounds. Our case demonstrates the need to consider the diagnosis of SCAD as a cause of ACS in men without any of the associated underlying conditions, especially in those patients with a recent history of lifting very heavyweight.

## Case presentation

A 58-year-old non-obese man with hypertension, asthma, and aspirin allergy presented with chest pain. The patient was a construction worker and reported being able to perform all of his duties that morning - including lifting machinery that was known to weigh between 200 and 250 pounds. He was in his usual state of health until lunchtime when he began experiencing pressure-like chest pain not relieved by rest.

In the emergency department, the patient endorsed pressure-like four out of ten chest pain, unlike anything he had ever experienced before. He denied nausea, vomiting, diaphoresis, orthopnea, or peripheral edema. He had not been taking any medications and had a medical history significant for an anaphylactic reaction to aspirin more than 20 years ago. He denied drug, tobacco, or alcohol use and had no family history of cardiovascular disease.

**Figure 1:**
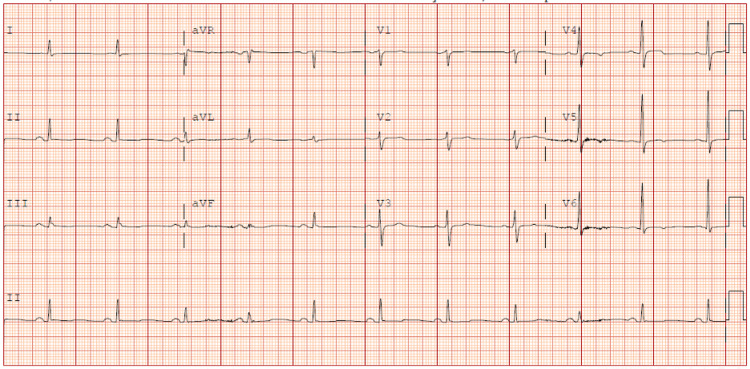
EKG demonstrating diffuse T-wave flattening and T-wave inversions in V4-6.

An EKG showed sinus bradycardia with diffuse T-wave flattening and T-wave inversions in V4-6 (Figure 1).

**Figure 2:**
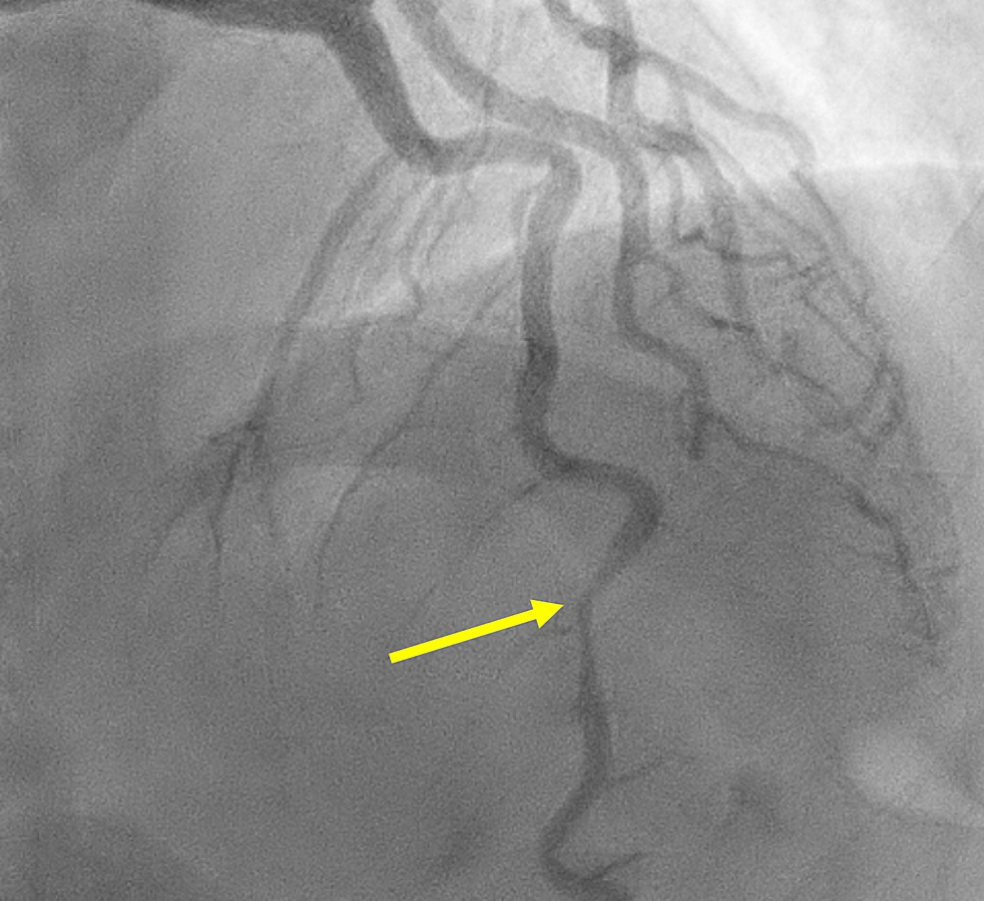
Left heart catheterization demonstrating coronary artery dissection of the mid-to distal-LAD (yellow arrow).

High sensitivity troponins were 2648 PG/ML (reference: less than 20 PG/ML), 3780 PG/ML at one hour, and 6270 PG/ML at three hours. Aspirin desensitization was performed with IV diphenhydramine and albuterol. Trans-thoracic echocardiography revealed an ejection fraction of 55% and no wall-motion abnormalities or diastolic dysfunction (possibly due to limited quality or because the degree of myocardial injury was not sufficient to visualize). Left heart catheterization demonstrated non-atherosclerotic coronary artery dissection of the mid-to distal-LAD (Figure 2).

Evaluation for fibromuscular dysplasia with CT angiography of the head and abdomen was negative. The patient was treated medically with aspirin, ticagrelor, atorvastatin, carvedilol, and amlodipine. He was discharged on these medications and given strict instructions not to lift more than 40 pounds. The patient is doing well ten months after the presentation.

## Discussion

Our case was unusual in that the patient was a man without an underlying condition associated with SCAD. In fact, roughly 90% of cases occur in women [5]. In at least 80% of cases, an underlying predisposing condition is present [5]. The most common conditions associated with SCAD are postpartum hormonal changes, multiparity, fibromuscular dysplasia, hormonal therapy, and systemic inflammatory conditions [5].

Acute stress preceding the onset of chest pain is reported in the majority of cases of SCAD: emotional stress is reported in 50.3% of cases and physical exertion in 28.9% of cases [2]. Other diseases associated with emotional stress, such as Takotsubo syndrome, should be ruled out after diagnosing SCAD. Lifting heavy weights has been associated with SCAD in both cohort studies and in case reports. In a six-year prospective and retrospective study at Vancouver General Hospital, 50 patients presented with SCAD, and three of them (6%) reported heavy weightlifting [6]. Two of these patients reported lifting greater than 50 pounds, while the other had lifted her 40-pound child into her car [6].

At least three other case reports describe patients who began experiencing chest pain after heavy lifting. One case involved an otherwise healthy 33-year-old lifting 30-pound weights at the gym when he began experiencing retrosternal chest pain [7]. Another man with no underlying health problems presented with chest pain after sexual activity after having repeatedly lifted greater than 50 pounds of weight two days prior and was found to have SCAD [8]. Finally, a 40-year-old woman was diagnosed with SCAD after experiencing chest pain that began while lifting a heavy garbage bag [9]. In our case, the patient reported lifting machinery that weighed between 200 and 250 pounds. To the best of our knowledge, this represents the heaviest weight reported in association with the development of SCAD.

The association between weightlifting and SCAD lends credence to the pathophysiological explanation that SCAD is driven by increased intraluminal pressure that causes shear stress and tears the intimal wall. Weightlifting is associated with transient extreme hypertension - one study demonstrated mean peak pressures among participants of 320/250 mmHg (one healthy participant had a peak pressure of 480/350 mmHg) [10]. The highest pressures were noted during double leg press - a movement similar to the one made by our patient in lifting heavy objects off the ground [10]. We suspect that the strength of association between SCAD and lifting weights increases at heavier weights. While insufficient data exist to prove such an association given the rarity of the condition, our case provides at least one example of the development of SCAD after lifting extremely heavyweight.

## Conclusions

SCAD is a rare condition that has classically been associated with peripartum women with certain diseases; however, it can occur in male patients without specifically associated comorbidities. The condition is managed medically. SCAD is associated with physical exertion and, specifically, lifting weight. Physicians should maintain an index of suspicion for SCAD in patients with anginal chest pain, especially when heavy lifting is reported.
